# CD4/CD8 Ratio Recovered as a Predictor of Decreased Liver Damage in Adults Infected With HIV: 16-Year Observational Cohort Study

**DOI:** 10.2196/45818

**Published:** 2024-01-09

**Authors:** Bingyu Liang, Rujing Sun, Yanyan Liao, Aidan Nong, Jinfeng He, Fengxiang Qin, Yanyun Ou, Jianhua Che, Zhenxian Wu, Yuan Yang, Jiao Qin, Jie Cai, Lijuan Bao, Li Ye, Hao Liang

**Affiliations:** 1 Guangxi Key Laboratory of AIDS Prevention and Treatment, School of Public Health, Guangxi Medical University Nanning China; 2 Biosafety III Laboratory, Life Science Institute Guangxi Medical University Nanning China; 3 HIV/AIDS prevention department Chongzuo Center for Disease Control and Prevention Chongzuo China

**Keywords:** AIDS, antiretroviral therapy, CD4/CD8, efavirenz, HIV, liver damage, lopinavir, nevirapine

## Abstract

**Background:**

As the life expectancy of individuals infected with HIV continues to increase, vigilant monitoring of non–AIDS-related events becomes imperative, particularly those pertaining to liver diseases. In comparison to the general population, patients infected with HIV experience a higher frequency of liver-related deaths. The CD4/CD8 ratio is emerging as a potential biomarker for non–AIDS-related events. However, few existing studies have been specially designed to explore the relationship between the CD4/CD8 ratio and specific types of non–AIDS-related events, notably liver damage.

**Objective:**

This study aimed to investigate the potential association between the CD4/CD8 ratio and the development of liver damage in a sizable cohort of patients infected with HIV receiving antiretroviral treatment (ART). Additionally, the study sought to assess the effectiveness of 3 antiretroviral drugs in recovering the CD4/CD8 ratio and reducing the occurrence of liver damage in this population.

**Methods:**

We conducted an observational cohort study among adults infected with HIV receiving ART from 2004 to 2020 in Guangxi, China. Propensity score matching, multivariable Cox proportional hazard, and Fine-Gray competing risk regression models were used to determine the relationship between the CD4/CD8 ratio recovered and liver damage.

**Results:**

The incidence of liver damage was 20.12% among 2440 eligible individuals during a median follow-up period of 4 person-years. Patients whose CD4/CD8 ratio did not recover to 1.0 exhibited a higher incidence of liver damage compared to patients with a CD4/CD8 ratio recovered (adjusted hazard ratio 7.90, 95% CI 4.39-14.21; *P*<.001; subdistribution hazard ratio 6.80, 95% CI 3.83-12.11; *P*<.001), findings consistent with the propensity score matching analysis (adjusted hazard ratio 6.94, 95% CI 3.41-14.12; *P*<.001; subdistribution hazard ratio 5.67, 95% CI 2.74-11.73; *P*<.001). The Efavirenz-based regimen exhibited the shortest time for CD4/CD8 ratio recovery (median 71, IQR 49-88 months) and demonstrated a lower prevalence of liver damage (4.18/100 person-years).

**Conclusions:**

Recovery of the CD4/CD8 ratio was associated with a decreased risk of liver damage in patients infected with HIV receiving ART, adding evidence for considering the CD4/CD8 ratio as a potential marker for identifying individuals at risk of non–AIDS-related diseases. An efavirenz-based regimen emerged as a recommended choice for recovering the CD4/CD8 ratio and mitigating the risk of liver damage.

## Introduction

In 2021, approximately 38.4 million individuals were infected with HIV, signifying a pressing global health issue. As of December 2021, about 28.7 million individuals worldwide have gained access to antiretroviral therapy (ART) [1]. The efficacy of ART in enhancing the survival of patients infected with HIV has led to a shift in the predominant causes of morbidity and mortality associated with HIV infection, moving away from opportunistic infections and AIDS-related neoplasms to non–AIDS-defining events, particularly cardiovascular and liver diseases, along with the accelerated progression to their advanced stages [2-4]. Specifically, the prevalence of liver-related deaths among patients infected with HIV exceeds that of the general population [5].

As awareness grows that individuals with HIV positive results will live to an age where liver damage is likely to occur, our understanding of the distinct features of liver injury in these patients, as well as the early diagnosis and therapy of liver disease, holds a pivotal role in enhancing clinical outcomes for patients with HIV positive results [6]. Consequently, there is an urgent need for surrogate indicators that can be used in clinical settings to identify individuals with vulnerable liver damage. The recovery of the CD4/CD8 ratio bears significant clinical relevance, and research has demonstrated that patients with a low ratio are characterized by altered T cell subsets and heightened CD8^+^ T cell activation, which is linked to the overall extent of SARS-CoV-2–specific T cell responses and an elevated risk of non–AIDS-related morbidity and mortality [7-10]. Importantly, the CD4/CD8 ratio is routinely recorded and readily available in clinical practice. Therefore, it could be easily used as a predictor of non-AIDS–related illnesses, including liver damage and others, provided that more data are acquired to substantiate its applicability.

Owing to an insufficient frequency of occurrences in each category, this study was not originally designed to explore the association between the CD4/CD8 ratio and specific non–AIDS-defining events, notably liver damage [9,11,12]. Furthermore, previous studies have evaluated the effectiveness of various ART regimens within drug classes on the recovery and elevation of the CD4/CD8 ratio. However, findings concerning the potential benefit of the CD4/CD8 ratio exhibit inconsistency. Variations between the ART drugs within each drug class might lead to differing impacts on the CD4/CD8 ratio, and the relationship between liver damage and these effects remains inadequately investigated [13].

The aim of this study was to determine whether the CD4/CD8 ratio, a readily obtainable metric in most clinical settings and frequently evaluated in patients infected with HIV exhibited an association with the occurrence of liver damage within a large cohort of patients infected with HIV, receiving ART. Additionally, we assessed a potential link between 3 distinct antiviral medications and the prevalence of liver damage, along with their impact on the CD4/CD8 ratio.

## Methods

### Participants and Study Setting

This was a retrospective cohort study. We collected the baseline and follow-up data from the National Free Antiretroviral Treatment Program database, a surveillance system that consistently records data on people living with HIV receiving free ART along with long-term follow-up care in China. We included all treatment-naïve people living with HIV aged 18 years or older who initiated therapy between November 15, 2004, and December 31, 2020, and were residents of Chongzuo City, Guangxi Province, China. Within the National Free Antiretroviral Treatment Program, routine monitoring of immunological parameters, including CD4^+^ and CD8^+^ cell counts, as well as other pertinent laboratory parameters and clinical treatment data such as bacterial-fungal infections and liver and kidney function, was performed.

### Statistical Analysis

Quantitative data were presented as the median (IQR), whereas categorical variables were given as the frequencies. To mitigate any potential bias stemming from treatment selection, the CD4/CD8 ratio recovered and unrecovered groups underwent a 1:2 propensity score matching (PSM) process. Similarly, the lopinavir-based, efavirenz-based, and NPV-based groups were subjected to a 1:1:1 PSM procedure. Following the methods reported previously [14], PSM analysis was used to match specific sociodemographic characteristics (such as sex, marital status, HIV transmission route, age at diagnosis, age at ART initiation, and BMI) between the groups with a recovered and unrecovered CD4/CD8 ratio. A caliper of 0.05 was used to ensure the alignment of characteristic factors between the 2 groups. Subsequently, the standardized mean differences (SMDs) and chi-square test were then used to assess the efficacy of the PSM, with SMDs exceeding 10% indicating substantial differences. Similarly, a caliper of 0.00001 was established for the ART regimen groups.

Subsequently, we used Cox proportional hazard regression models to estimate the relationship between CD4/CD8 ratio recovery and the occurrence of liver damage. To determine whether CD4/CD8 ratio recovery is linked to a reduced risk of liver damage when competing risk of mortality is accounted for, we also used Fine-Gray competing risk regression models [15,16]. Using these same methodologies, we assessed the association between the ART regimen and the incidence of liver damage with the intention of enhancing clinical decision-making. A set of factors were selected as covariates: CD4/CD8 ratio recovery, ART regimens, sex, marital status, age at HIV diagnosis, age at ART initiation, HIV transmission route, WHO (World Health Organization) HIV disease stage at baseline, BMI at baseline, CD4^+^ lymphocyte count at baseline, CD8^+^ lymphocyte count at baseline, CD4/CD8 ratio at baseline, cytomegalovirus (CMV) infection, aspartate aminotransferase (AST) at baseline, alanine aminotransferase (ALT) at baseline, and total bilirubin (TBIL) at baseline.

To visually represent the hazard ratio before and after PSM, a forest plot was generated. Additionally, as a sensitivity analysis to assess the potential for residual confounding, we calculated end point E-values [17]. Statistical significance was assessed at α=.05, and all analyses were 2-tailed. The statistical analyses were performed with R (version 4.1.0; R foundation for Statistical Computing) and SPSS Statistics (version 26.0; IBM Corp). The “MatchIt” R package (Noah Greifer) was used for PSM, while the “survival,” “cmprsk,” and “splines” R packages for Fine-Gray competing risk regression.

### Ethical Considerations

This study was approved by the Human Research Ethics Committee of Guangxi Medical University (2019-SB-102), and it was carried out in accordance with the Helsinki Declaration. In this study site, when the patients first attended the HIV prevention department of Center of Disease Control and the HIV care clinic of the hospital, they were informed that their anonymized data could be used for research. As the data collection and extraction were anonymized, individual informed consent was not needed for this study. An informed consent waiver was approved by the institutional review board of Guangxi Medical University. Participants were compensated 50 RMB (US $7.14) or a free blood routine test for the first visit and each follow-up.

We identified liver damage as the primary end point during the follow-up period. This was delineated by the combined assessment of 3 liver function parameters: AST, ALT, and TBIL. We established reference values of AST=40 unit liter, ALT=40 unit liter, and TBIL=20 mol/L as the upper limits of normality (ULN) based on the division of AIDS toxicity guidelines. All values surpassing these thresholds were classified as abnormal and categorized into different grades of liver enzyme elevation (LEE) or total bilirubin elevation (TBE) as defined by the guidelines. Specifically, grade I-IV LEE was defined as elevations ranging from 1-2.5, 2.5-5, 5-10, to more than 10 times the ULN, while grade I-IV TBE was determined by elevations of 1-1.5, 1.5-2.5, 2.5-5, or more than 5 times the ULN. Participants with no LEE or TBE or only grade I LEE or TBE were categorized into the normal liver function group, while those with grade II, III, or IV LEE or TBE were classified into the liver damage group.

In addition to 2 nucleoside reverse transcriptase inhibitors, cART (combination antiretroviral treatment) was defined as a combination regimen that included a nonnucleoside reverse transcriptase inhibitor, protease inhibitor, or integrase strand transfer inhibitor. The ART regimens were categorized based on the primary medication used within the initial cART regimen. Specifically, these categories encompassed lopinavir from the protease inhibitor class and nevirapine and efavirenz from the nonnucleoside reverse transcriptase inhibitor class.

The baseline encompassed various clinical indices before the initiation of ART. Follow-up persisted until the earlier occurrence of the following events: liver damage, death, or the final clinical visit recorded at the end of the study.

The inclusion criteria for patients in this study were as follows: (1) having normal liver function parameters at baseline (including normal serum ALT, AST, and TBIL), (2) having at least 2 records of CD4/CD8 ratio, and (3) receiving combination ART regimens. Patients who met any of the following criteria were excluded from the study: (1) absence of baseline CD4^+^ or CD8^+^ lymphocyte count data, (2) absence of records for any of the 3 liver function markers (AST, ALT, or TBIL), (3) lack of normal liver function at baseline, (4) presence of only 1 CD4/CD8 ratio record, and (5) were treated with regimens other than cART.

The recovery of the CD4/CD8 ratio was defined as presence of 2 consecutive values ≥1.0 (with an interval between 2 consecutive records ranging from 180 days to less than 2 years) during the follow-up period [18]. Individuals meeting this criterion were defined as the recovered group, whereas those not meeting the criterion were grouped as the unrecovered group.

## Results

**Figure 1:**
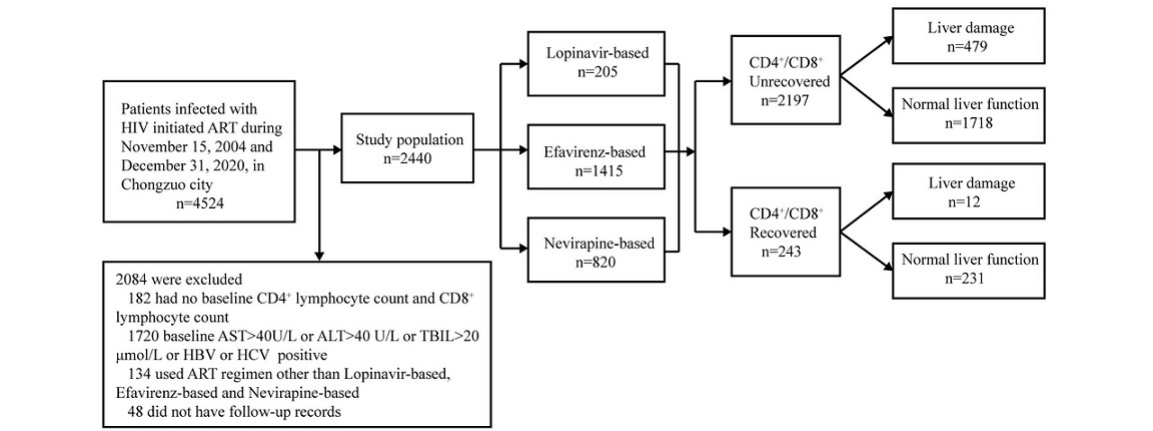
Cohort enrollment criteria. ALT: alanine aminotransferase; ART: antiretroviral treatment; AST: aspartate aminotransferase; HBV: hepatitis B virus; HCV: hepatitis C virus; TBIL: total bilirubin.

We identified a total of 2440 patients with HIV who met the inclusion and exclusion criteria. The patient flow for inclusion in the analysis is presented in Figure 1. Among these eligible patients, the cumulative person-years of follow-up amounted to 9963, with a median of 4 (IQR 1-7) person-years. Within the cohort, 1516/2440 (62.13%) individuals were male, while 1616/2440 (66.23%) were married or cohabiting. The majority of patients were diagnosed with HIV (1043/2440, 42.75%) or initiated ART (1074/2440, 44.02%) at the age of 50 years or more. Heterosexual transmission accounted for 93.40% (2279/2440) of HIV acquisition, and 33.40% (815/2440) had progressed to WHO HIV clinical stage I disease at baseline. Regarding baseline characteristics, 53.12% (1296/2440) of patients had a normal BMI ranging from 18.5 to 23.9 kg/m^2^. Furthermore, 51.19% (1249/2440) of patients exhibited a CD4^+^ lymphocyte count below 200 cells/μL, while 50.08% (1222/2440) of them had a CD8^+^ lymphocyte count ≤800 cells/μL. Notably, 68.89% (1681/2440) of patients displayed a CD4/CD8 ratio below 0.30 at baseline. Additionally, 0.70% (17/2440) of patients showed anti-CMV Immunoglobulin G antibodies (overall 17/2440, 0.70%; when only patients with an available CMV serology were considered: 17/2275, 0.74%; Table 1).

As illustrated in Table S1 in Multimedia Appendix 1 , 9.96% (243/2440) of patients achieved CD4/CD8 ratio recovery when the cutoff point for CD4/CD8 ratio recovery was set at 1. To determine the most informative cutoff point, 3 distinct thresholds were tested. Fine-Gray competing risk regression analysis indicated that the highest subdistribution hazard ratio (sHR) was observed (sHR 6.80, 95% CI 3.83-12.11) when the cutoff point was set at 1.0. This value notably exceeded the sHR for a cutoff of 0.5 (sHR 5.93, 95% CI 4.63-7.58) and a cutoff of 1.2 (sHR 4.90, 95% CI 2.51-12.11). Consequently, a CD4/CD8 ratio recovery cutoff threshold of 1.0 was selected for subsequent analysis.

Tables S2 and S3 in Multimedia Appendix 1 showed the results of PSM, revealing that 480 patients with recovered CD4/CD8 ratios and 242 patients with unrecovered CD4/CD8 ratios were included. The matched participants from the 3 ART regimen groups were merged, resulting in a total of 501 patients (167 in the lopinavir-based group, 167 in the efavirenz-based group, and 167 in the nevirapine-based group). After matching, these demographic characteristics no longer exhibited statistically significant disparities across groups (SMDs<10% and *P*>.05).

**Figure 2:**
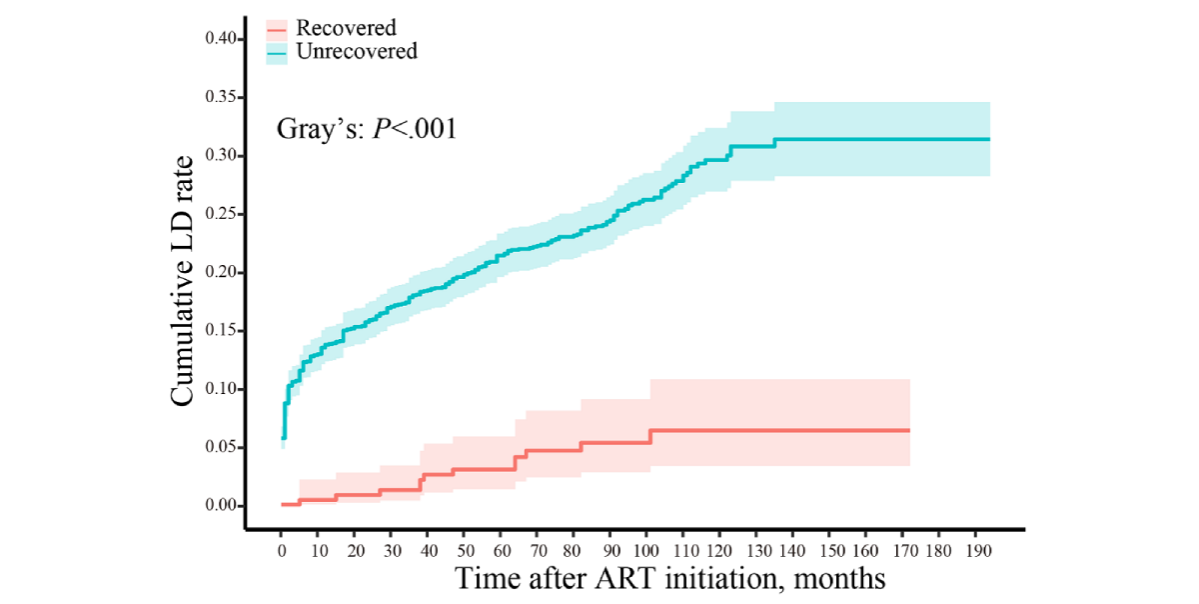
Unadjusted cumulative incidence of liver damage among adults infected with HIV receiving antiretroviral therapy, accounting for the competing risk of death. ART: antiretroviral treatment; LD: liver damage.

**Figure 3:**
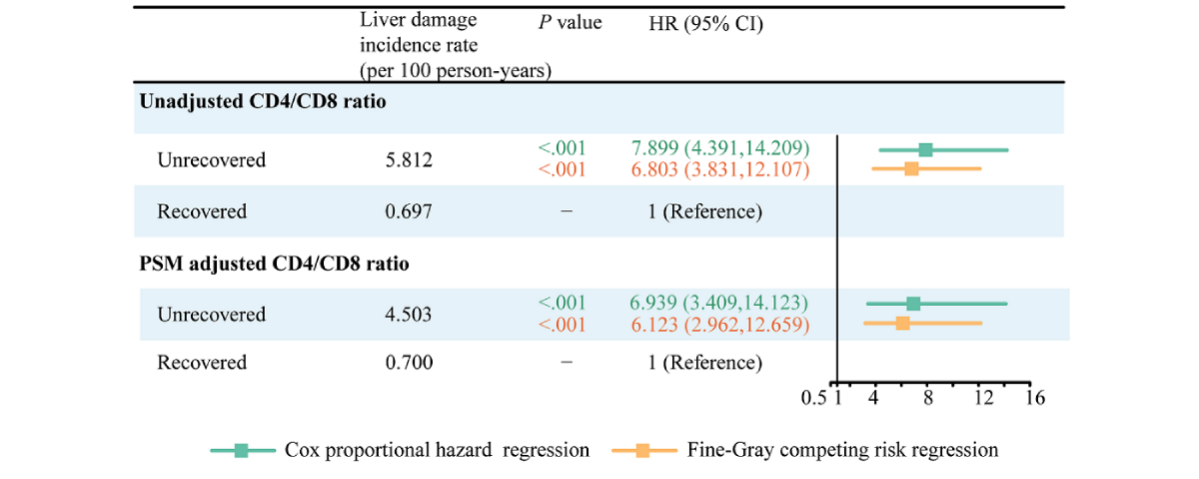
Forest plots of multivariable Cox proportional hazard regression and Fine-Gray competing risk regression analysis of the effect of CD4/CD8 ratio recovery on liver damage among patients with HIV receiving antiretroviral treatment (ART). Hazard ratio adjusted by CD4/CD8 ratio recovery, ART regimen, sex, marital status, age at HIV diagnosis, age at ART initiation, HIV transmission route, World Health Organization HIV disease stage, BMI at baseline, CD4+ lymphocyte count at baseline, CD8+ lymphocyte count at baseline, CD4/CD8 ratio at baseline, cytomegalovirus infection, aspartate aminotransferase at baseline, alanine aminotransferase at baseline, and total bilirubin at baseline. HR: hazard ratio; PSM: propensity score matching.

Figure 2 shows the cumulative incidence curve, illustrating the unadjusted incidence of liver damage in both the recovered and unrecovered CD4/CD8 ratio groups. The Fine-Gray test revealed a substantially higher cumulative incidence of liver damage in individuals with a non-recovered CD4/CD8 ratio compared to those with a recovered ratio over the 16-year follow-up period (*P*<.001). Upon conducting multivariable analysis, both Cox proportional hazard regressions and Fine-Gray competing risk regressions (Figure 3) indicated that a nonrecovered CD4/CD8 ratio was associated with a heightened risk of liver damage both before (adjusted hazard ratio [aHR] 7.899, 95% CI 4.391-14.209; *P*<.001; sHR 6.803, 95% CI 3.831-12.107; *P*<.001) and after PSM (aHR 6.939, 95% CI 3.409-14.123; *P*<.001; sHR 6.123, 95% CI 2.962-12.659; *P*<.001). The aHR and sHR values for the other covariates are presented in Table S4 in Multimedia Appendix 1. Additionally, the E-value analysis revealed that an unmeasured confounding factor would need to exhibit a minimum association (aHR) of 6.17 (95% CI 2.55-9.79) with liver damage, considering the measured covariates, to challenge the observed outcome.

**Figure 4:**
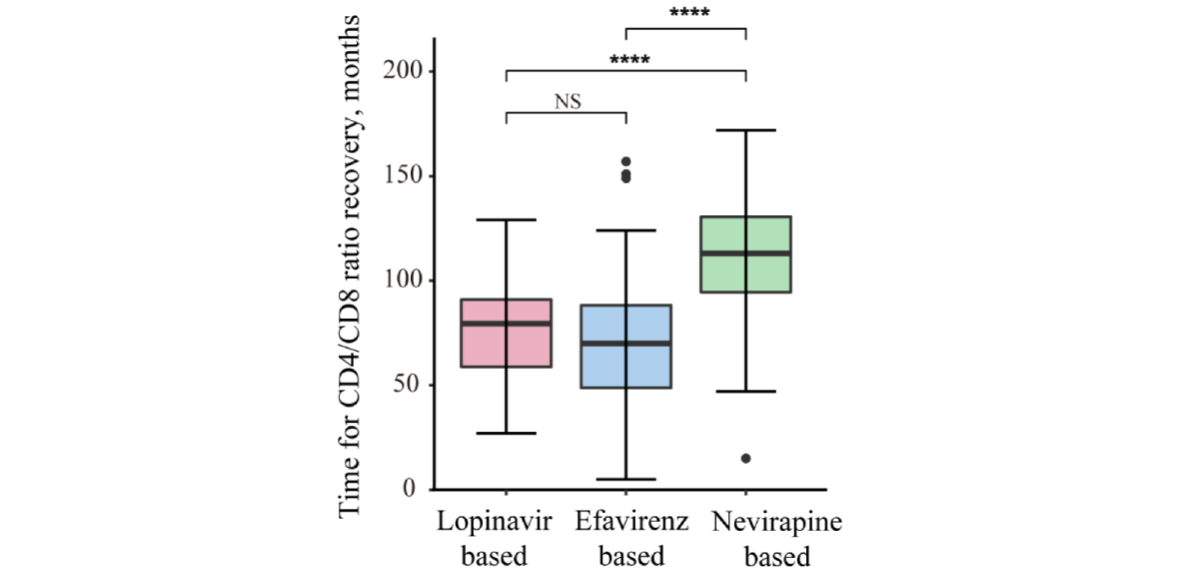
Time for CD4/CD8 ratio recovery among different antiretroviral treatment regimen groups. NS: not statistically significant. ****P<.0001.

The CD4/CD8 ratio recovered rate exhibited no significant differences among the 3 ART treatment groups (*χ*^2^= 0.04; *P*=.98; *v*=2). Especially, the CD4/CD8 ratio recovery rate was 10.1% for the nevirapine-based group, 10% for the efavirenz-based group, and 9.9% for the lopinavir-based group (Table S5 in Multimedia Appendix 1). In addition, there was a notable disparity in the time required for CD4/CD8 ratio recovery (defined as the interval between cART initiation and the attainment of CD4/CD8 ratio recovery, measured in months) across the 3 ART regimen groups (*P*=.01). The nevirapine-based group had the longest duration for recovery (113 months, IQR 95-131 months), substantially surpassing the efavirenz-based (71 months, IQR 49-88 months) and lopinavir-based (77 months, IQR 59-91 months) groups (Figure 4).

**Figure 5:**
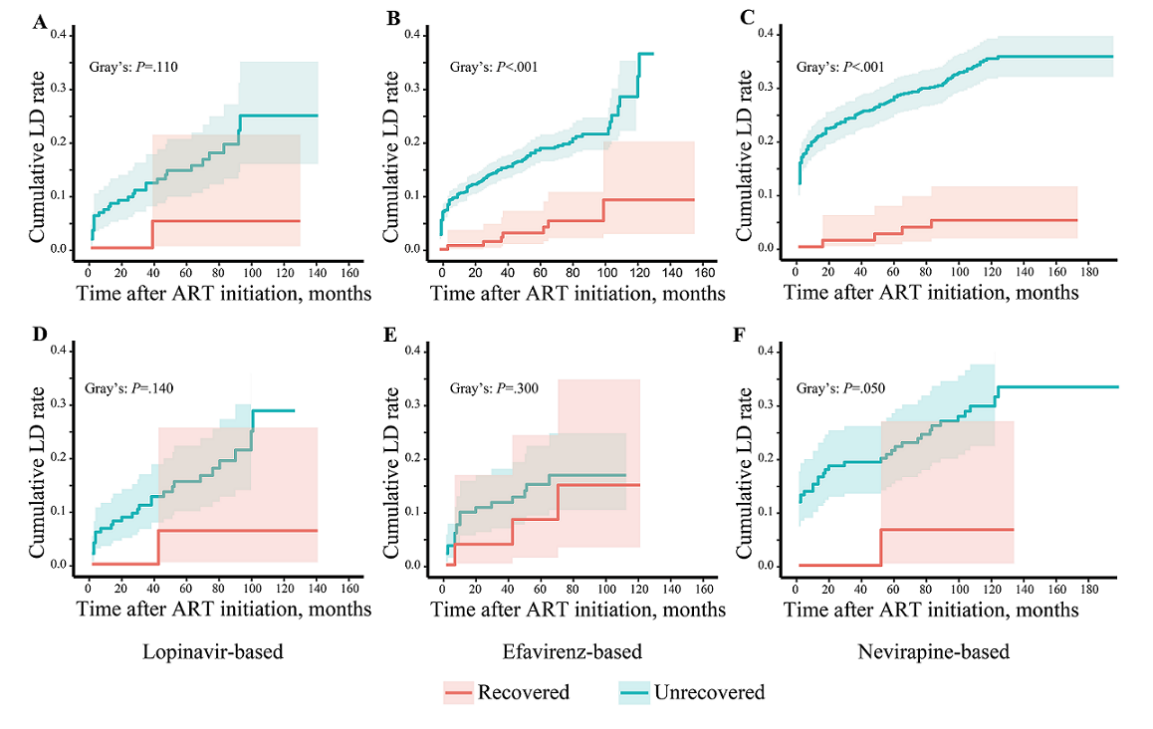
Cumulative incidence of liver damage (LD) for patients with HIV receiving antiretroviral treatment (ART), grouped by ART regimen. (A) The whole research population of patients who were treated with a lopinavir-based regimen. (B) The whole research population of patients who were treated with an efavirenz-based regimen. (C) The whole research population of patients who were treated with a nevirapine-based regimen. (D) The propensity score matching (PSM) of patients treated with a lopinavir-based regimen. (E) PSM patients treated with an efavirenz-based regimen. (F) PSM patients treated with a nevirapine-based regimen. The statistical significance was measured by the Fine-Gray test. LD: liver damage.

Through the use of stratified analysis based on different ART regimens, the relationship between CD4/CD8 ratio recovery and liver damage was assessed across 3 distinct subgroups characterized by competing mortality risks. In the nevirapine-based regimen, it was evident that the cumulative incidence of liver damage was significantly higher in the CD4/CD8 ratio unrecovered group compared to the recovered group, both before (Figure 5C) and after PSM (Figure 5F). However, within the group administered lopinavir and efavirenz regimens, no notable discrepancy in liver damage incidence was observed after PSM (Figures 5A, 5D, and 5F). Notably, patients subjected to the nevirapine-based regimen and exhibiting an unrecovered CD4/CD8 ratio displayed the highest liver damage incidence rate (7/100 person-years), a notably elevated figure in comparison to CD4/CD8 ratio recovered patients (Table S6 in Multimedia Appendix 1).

**Figure 6:**
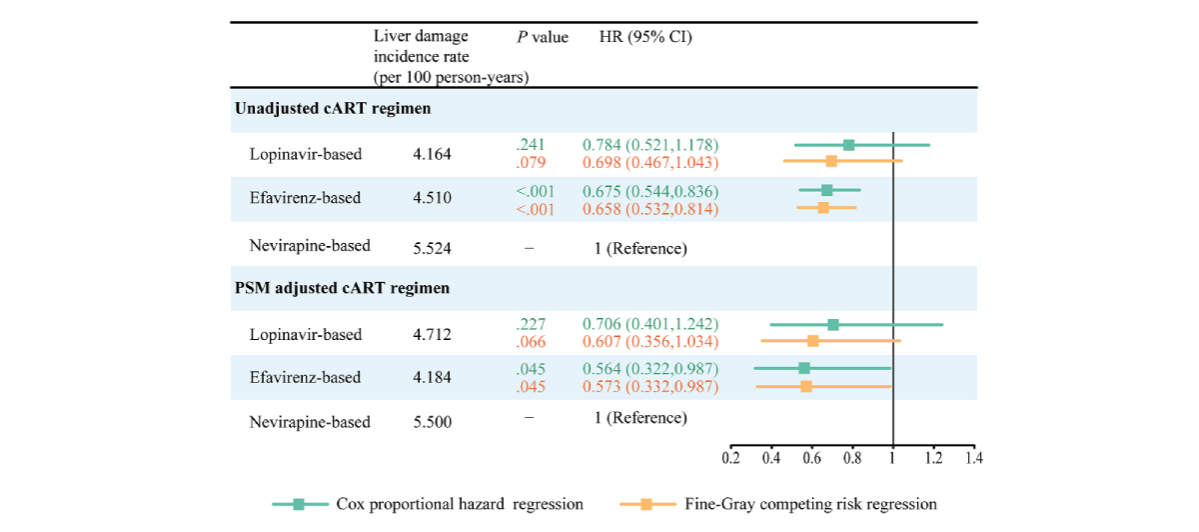
Forest plots of multivariable Cox proportional hazard regression and Fine-Gray competing risk regression analysis of the effect of antiretroviral treatment (ART) regimen on liver damage among patients with HIV receiving ART. cART: combination antiretroviral treatment; HR: hazard ratio; PSM: propensity score matching.

As shown in Figure 6, after being modified for a specified group of factors through forward-selection, both the Cox proportional hazard time-to-event regressions and Fine-Gray competing risk regressions (Figure 6) indicated that the efavirenz-based regimen was associated with a notably reduced incidence of liver damage than the nevirapine-based regimen. This distinction held true both before (aHR 0.675, 95% CI 0.544-0.836; *P*<.001; sHR 0.658, 95% CI 0.532-0.814; *P*<.001) and after PSM (aHR 0.564, 95% CI 0.322-0.987; *P*=.045; sHR 0.573, 95% CI 0.332-0.987; *P*=.045).

**Table 1:** Sociodemographic characteristics of patients with HIV receiving antiretroviral treatment (ART; N=2440).

Variable			Study population, n (%)
**Sex**			
	Male		1516 (62.13)
	Female		924 (37.87)
**Marital status**			
	Married or cohabitation	1616 (66.23)	
	Divorced, separated, or widowed	493 (20.21)	
**HIV** **transmission** **route**			
	Heterosexual contact		2279 (93.40)
	Homosexual contact		37 (1.52)
	Blood or plasma transfusion		124 (5.08)
**Age at diagnosis (years)**			
	<30		442 (18.12)
	30-49		955 (39.14)
	≥50		1043 (42.75)
**Age at ART initiation (years)**			
	<30		386 (15.82)
	30-49		980 (40.16)
	≥50		1074 (44.02)
**BMI at baseline, kg/m^2^**			
	<18.5		585 (23.98)
	18.5-23.9		1296 (53.12)
	24-27.9		169 (6.93)
	≥28		27 (1.11)
	Unknown		363 (14.88)
**WHO^a^ HIV disease stage at baseline**			
	I		815 (33.40)
	II		592 (24.26)
	III		434 (17.79)
	IV		492 (20.16)
	Unknown		107 (4.39)
**CD4^+^ lymphocyte count at baseline, cells/μL**			
	<200		1249 (51.19)
	200-349		813 (33.32)
	≥350		378 (15.49)
**CD8^+^ lymphocyte count at baseline, cells/μL**			
	<800		1222 (50.08)
	800-1499		913 (37.42)
	≥1500		305 (12.50)
**CD4/CD8 ratio at baseline**			
	<0.3		1681 (68.89)
	0.3-0.59		670 (27.46)
	≥0.6		89 (3.65)
**Cytomegalovirus infection**			
	No		2258 (92.54)
	Yes		17 (0.70)
	Unknown		165 (6.76)

^a^WHO: World Health Organization.

## Discussion

### Principal Results

This study represents the first comprehensive large cohort study conducted in a real-world setting with long-term follow-up to examine the association between CD4/CD8 ratio recovery and the risk of liver damage among patients infected with HIV receiving ART. Our findings underscored the notable incidence of liver damage and its strong association with the unrecovered CD4/CD8 ratio. Furthermore, we observed that participants treated with an efavirenz-based regimen exhibited a diminished incidence of liver damage and an unrecovered CD4/CD8 ratio in comparison to those administered with the nevirapine-based regimen. Given that this study centered on individuals with HIV with initially normal liver function, with the end point defined as the occurrence of liver damage, these results bear significant implications for comprehending the interrelation between the CD4/CD8 ratio and non–AIDS-defining events in patients with HIV. Thus, this study contributes substantially to the body of research in this field.

### Comparison With Previous Work

In this study, liver damage was observed in 20% (491/2440) of the participants, a prevalence in line with earlier studies that reported figures ranging from 12% to 27% [19,20]. However, it is noteworthy that the highest incidence of liver damage reported in this study was 48%, occurring within the initial 3 months following the commencement of cART. This rate is both higher and faster than what was observed in a previous study (35% at 9 months) [19]. Nearly half of the patients in this cohort were older adults, aligning with the prevalent demographic trend among patients in Guangxi province [21,22]. Indeed, the elevated incidence of liver damage can be attributed to the advanced age of the patient cohort. The higher incidence underscores the substantial burden of liver disease within these individuals and the early onset of liver function impairment upon initiating cART, accentuating the significance of the initial cART regimen.

CD4/CD8 ratio recovery exhibited a robust correlation with a notable reduction in the incidence of liver damage, a relationship confirmed by both Cox regression and PSM analysis, methodologies designed to mitigate bias stemming from potential confounding variables. Notably, the observed risk reduction in liver damage remained consistent across various ratio thresholds, even when accounting for the presence of competing mortality risks. Despite variations in analytic approaches and threshold values, these findings align with results reported by Mussini et al [9], which demonstrated that a diminished CD4/CD8 ratio following ART initial independently contributed to an escalated risk of severe non–AIDS-defining events.

Furthermore, a study conducted on individuals coinfected with the hepatitis C virus and HIV demonstrated a close correlation between liver damage, assessed through transaminase levels, and the CD4/CD8 ratio [23]. These findings align with other research that has established an association between a low CD4/CD8 ratio and markers of liver health, such as liver stiffness and fibrosis [24]. Additionally, this ratio has been linked to unfavorable outcomes in smaller, often single-center cohorts [7,25,26]. However, contrasting results have been reported. For instance, a Canadian cohort study, after adjusting for variables including age and HIV RNA levels, found the CD4/CD8 ratio did not offer additional short-term prognostic value for clinical outcomes [27]. A diminished or inverted CD4/CD8 ratio reflects sustained immunological activation and is associated with immunodiscordance and the persistent inflammation that is characteristic of HIV infection [25,28,29].

Besides HIV-induced hepatitis, drug-induced hepatotoxicity constitutes a significant cause of liver damage. A post hoc analysis of previous studies revealed that an efavirenz-based ART regimen exhibited a more substantial improvement in the CD4/CD8 ratio compared to other frequently used ART regimens [30,31]. In this study, we further explored the relationship between the extent and duration of CD4/CD8 ratio recovery and the incidence of liver damage in the 3 main ART regimens. Our findings indicated that there were no significantly differences in the rates of CD4/CD8 ratio recovery at a cutoff of 1.0 across the three ART regimen groups. However, it is worth highlighting that the efavirenz-based regimen was associated with a significant shorter period until CD4/CD8 recovery compared to the nevirapine-based regimen. Notably, users of efavirenz and lopinavir exhibited a greater enhancement in the CD4/CD8 ratio than nevirapine users, a result consistent with findings from a different research cohort [32].

In addition, this study demonstrated a consistent correlation between CD4/CD8 ratio recovery and reduced liver damage in nevirapine-based regimen groups, as validated by PSM analysis. This conclusion is further supported by the observation that patients receiving nevirapine-based therapy exhibited the longest duration to achieve CD4/CD8 ratio recovery in this study. The outcomes of this study hold clinical significance, particularly within resource-limited settings, and underscore the need for careful consideration of CD4/CD8 ratio recovery in this population to mitigate the risk of liver damage. This recommendation holds particular weight given the lifelong duration of ART regimens, where drug toxicity assumes paramount importance.

### Limitations

This research has several limitations that warrant consideration. First, as a retrospective clinical study, the presence of unmeasured confounding variables is a potential concern. Second, the assessment of antiretroviral drug adherence, a factor that could affect the CD4/CD8 ratio recovery, was not feasible within the scope of this study. Third, this study lacked control over the use of medications other than ART regimens, some of which could be associated with hepatotoxicity. Last, in absence of viral suppression data in our cohort precludes us from definitively ascertaining occurrences of virological failure. Despite these limitations, the strength of our research lies in its substantial patient enrollment and robust multivariate analysis. Notably, the application of E-value analysis underscores that a potent confounding variable would be required to reverse the observed findings. An additional advantage is the use of competing-risk analysis, which provides unbiased estimations of cumulative events by accounting for death as a competing event. The stringent baseline liver function inclusion criteria further contribute to the study’s strengths; however, it should be acknowledged that selection bias could be present, given that a portion of patients were excluded due to these rigorous criteria.

### Conclusions

This study had clinical and public health significance and offered additional evidence that the recovery of the CD4/CD8 ratio was associated with a lower risk of liver damage compared to ratio inversion. Notably, patients with HIV treated with efavirenz-based and lopinavir-based regimens exhibited a more pronounced increase in the CD4/CD8 ratio and a lower incidence of liver damage in contrast to those receiving nevirapine-based regimens. These findings highlight the importance of ongoing monitoring of the CD4/CD8 ratio and prompt intervention in the event of ratio inversion, particularly for individuals under nevirapine-based ART regimens.

## Appendix

Multimedia Appendix 1 Supplementary tables.

## Abbreviations

aHR: adjusted hazard ratio
ALT: alanine aminotransferase
ART: antiretroviral treatment
AST: aspartate aminotransferase
cART: combination antiretroviral treatment
CMV: cytomegalovirus
LEE: liver enzyme elevation
PSM: propensity score matching
sHR: subdistribution hazard ratio
SMD: standardized mean difference
TBE: total bilirubin elevation
TBIL: total bilirubin
ULN: upper limits of normality
WHO: World Health Organization
