# Supplementary material for: CD4/CD8 Ratio Recovered as a Predictor of Decreased Liver Damage in Adults Infected With HIV: 16-Year Observational Cohort Study
Source: JMIR Public Health Surveill. 2024 Jan 9;10:e45818. doi: 10.2196/45818 (PMC10806443; doi:10.2196/45818)
Supplement: Multimedia Appendix 1 [file publichealth_v10i1e45818_app1.docx]

Table S1. Effect of the CD4/CD8 ratio recovery at different cutoff point on liver damage among HIV patients receiving ART.^a^

|  | Total patients  n (%) | Patients who developed liver damage n (%) | sHR ^a^ (95%CI) | *P* |
| --- | --- | --- | --- | --- |
|  |  |  |  |  |
| **CD4/CD8 ratio recovery at a cutoff of 0.5** |  |  |  |  |
| recovered | 950(0.39) | 89(9.37) | 1 (Reference) | - |
| unrecovered | 1490(0.61) | 402(26.98) | 5.93(4.63-7.58) | <.001 |
| **CD4/CD8 ratio recovery at a cutoff of 1.0** |  |  |  |  |
| recovered | 243(0.10) | 12(4.94) | 1 (Reference) | - |
| unrecovered | 2197(0.90) | 479(21.80) | 6.80(3.83-12.11) | <.001 |
| **CD4/CD8 ratio recovery at a cutoff of 1.2** |  |  |  |  |
| recovered | 143(0.06) | 9(6.29) | 1 (Reference) | - |
| unrecovered | 2297(0.94) | 482(20.98) | 4.90(2.52-9.52) | <.001 |

^a^sHR, sub-distribution HR.

.

Table S2. Baseline demographic characteristics of subgroup.^a^

|  | CD4/CD8 ratio recovery PSM adjusted^a^, n (%) | | |  | cART regime PSM adjusted^a^, n (%) | | | |  |
| --- | --- | --- | --- | --- | --- | --- | --- | --- | --- |
|  | Overall | Unrecovered | Recovered | *P* | Overall | LPV | EFV | NVP | *P* |
|  |  |  |  |  |  |  |  |  |  |
| Total No. | 722 | 480 | 242 |  | 501 | 167 | 167 | 167 |  |
| Sex |  |  |  | .953 |  |  |  |  | .880 |
| Male | 339（47） | 225（46.9） | 114（47.1） |  | 259(51.7) | 85(50.9) | 89(53.3) | 85(50.9) |  |
| Female | 383（53） | 255（53.1） | 128（52.9） |  | 242(48.3) | 82(49.1) | 78(46.7) | 82(49.1) |  |
| Marital status |  |  |  | .969 |  |  |  |  | .716 |
| Unmarried | 44（6.1） | 30（6.3） | 14（5.8） |  | 19(3.8) | 7(4.2) | 5(3) | 7(4.2) |  |
| Married/cohabitation | 524（72.6） | 348（72.5） | 176（72.7） |  | 368(73.5) | 125(74.9) | 118(70.7) | 125(74.9) |  |
| Divorced/separated/widowed | 154（21.3） | 102（21.3） | 52（21.5） |  | 114(22.8) | 35(21) | 44(26.3) | 35(21) |  |
| HIV transmission route |  |  |  | .127 |  |  |  |  | .903 |
| Heterosexual contact | 706（97.8） | 470（97.9） | 236（97.5） |  | 491(98) | 164(98.2) | 163(97.6) | 164(98.2) |  |
| Homosexual contact | 2（0.3） | 0（0） | 2（0.8） |  | 0(0) | 0(0) | 0(0) | 0(0) |  |
| Blood/plasma transfusion | 14（1.9） | 10（2.1） | 4（1.7） |  | 10(2) | 3(1.8) | 4(2.4) | 3(1.8) |  |
| Age at diagnosis,year |  |  |  | .978 |  |  |  |  | .424 |
| <30 | 104（14.4） | 70（14.6） | 34（14） |  | 85(17) | 28(16.8) | 29(17.4) | 28(16.8) |  |
| 30～49 | 257（35.6） | 170（35.4） | 87（36） |  | 59(11.8) | 23(13.8) | 13(7.8) | 23(13.8) |  |
| ≥50 | 361（50） | 240（50） | 121（50） |  | 357(71.3) | 116(69.5) | 125(74.9) | 116(69.5) |  |
| Age at ART initiation, year |  |  |  | .938 |  |  |  |  | .802 |
| <30 | 91（12.6） | 62（12.9） | 29（12） |  | 80(16) | 28(16.8) | 24(14.4) | 28(16.8) |  |
| 30～49 | 264（36.6） | 175（36.5） | 89（36.8） |  | 64(12.8) | 23(13.8) | 18(10.8) | 23(13.8) |  |
| ≥50 | 367（50.8） | 243（50.6） | 124（51.2） |  | 357(71.3) | 116(69.5) | 125(74.9) | 116(69.5) |  |
| BMI, kg/m2 # |  |  |  | .988 |  |  |  |  | .485 |
| <18.5 | 120（16.6） | 79（16.5） | 41（16.9） |  | 149(29.7) | 51(30.5) | 47(28.1) | 51(30.5) |  |
| 18.5–23.9 | 411（56.9） | 275（57.3） | 136（56.2） |  | 281(56.1) | 92(55.1) | 97(58.1) | 92(55.1) |  |
| 24–27.9 | 63（8.7） | 43（9） | 20（8.3） |  | 42(8.4) | 13(7.8) | 16(9.6) | 13(7.8) |  |
| ≥28 | 8（1.1） | 5（1） | 3（1.2） |  | 2(0.4) | 0(0) | 2(1.2) | 0(0) |  |
| Unknown | 120（16.6） | 78（16.3） | 42（17.4） |  | 27(5.4) | 11(6.6) | 5(3) | 11(6.6) |  |

Abbreviations: PSM, Propensity score matching; HIV, human immunodeficiency virus; ART, antiretroviral treatment; BMI, body mass index.

^a^The propensity score used for PSM in this table was based on all characteristics listed in this table.

Table S3. Standard mean differences (SMDs) for subgroups before and after PSM.

|  | CD4/CD8 ratio recovery ^a^ | | ART regime PSM adjusted^a^ | | | |
| --- | --- | --- | --- | --- | --- | --- |
|  | Recovered group & Unrecovered group | | LPV-based group & EFV-based group | | LPV-based group & NVP-based group | |
|  | SMD before PSM adjusted | SMD after PSM adjusted | SMD before PSM adjusted | SMD after PSM adjusted | SMD before PSM adjusted | SMD after PSM adjusted |
| **Sex** | 0.3 | -0.012 | 0.267 | 0 | 0.405 | 0 |
| **Marital status** | 0.209 | 0.008 | 0.282 | 0 | 0.318 | 0 |
| **HIV transmission route** | -0.312 | 0 | -0.116 | 0 | -0.262 | 0 |
| **Age at diagnosis, year** | 0.173 | 0.003 | 0.199 | 0 | 0.445 | 0 |
| **Age at ART initiation, year** | 0.181 | 0.015 | 0.181 | 0 | 0.463 | 0 |
| **BMI, kg/m2** | 0.141 | 0.006 | -0.026 | 0 | -0.899 | 0 |

Abbreviations: PSM, Propensity score matching; HIV, human immunodeficiency virus; ART, antiretroviral treatment; BMI, body mass index.

^a^The propensity score used for PSM in this table was based on all characteristics listed in this table.

Table S4. Adjusted hazard based on multivariable Cox proportional hazard regression and Fine-Gray competing risk regression of the effect of CD4/CD8 ratio recovery on liver damage among subgroup of Study cohort.

|  | Cox proportional hazard regression | | | Fine-Gray competing risk regression | | |
| --- | --- | --- | --- | --- | --- | --- |
| Characteristic | *P* | aHR | 95%CI | *P* | sHR | 95%CI |
| **Sex** | .036 | 1.258 | (1.016, 1.559) | .13 | 0.842 | (0.676, 1.049) |
| **Marital status** |  |  |  |  |  |  |
| Unmarried | .354 | 0.847 | (0.597, 1.203) | .38 | 1.164 | (0.831, 1.630) |
| Married/cohabitation | .402 | 0.904 | (0.713, 1.145) | .77 | 1.036 | (0.820, 1.308) |
| Divorced/separated/widowed | Ref | _ | _ | Ref | _ | _ |
| **HIV transmission route** |  |  |  |  |  |  |
| Heterosexual contact | .007 | 0.639 | (0.461, 0.886) | .00079 | 1.674 | (1.239, 2.261) |
| Homosexual contact | .266 | 0.583 | (0.225, 1.51) | .35 | 1.566 | (0.605, 4.048) |
| Blood/plasma transfusion | Ref | _ | _ | Ref | _ | _ |
| **Age at diagnosis,year** |  |  |  |  |  |  |
| <30 | .792 | 1.149 | (0.41, 3.216) | .73 | 0.847 | (0.327, 2.192) |
| 30～49 | .415 | 1.409 | (0.618, 3.214) | .34 | 0.694 | (0.328, 1.466) |
| ≥50 | Ref | _ | _ | Ref | _ | _ |
| **Age at ART initiation, year** |  |  |  |  |  |  |
| <30 | .938 | 0.959 | (0.338, 2.72) | .98 | 0.985 | (0.380, 2.551) |
| 30～49 | .91 | 0.954 | (0.42, 2.167) | .98 | 1.008 | (0.676, 1.050) |
| ≥50 | Ref | _ | _ | Ref | _ | _ |
| **BMI at baseline, kg/m2 #** |  |  |  |  |  |  |
| <18.5 | .324 | 0.857 | (0.632, 1.164) | .042 | 1.362 | (1.011, 1.834) |
| 18.5–23.9 | .146 | 0.819 | (0.625, 1.072) | .027 | 1.344 | (1.034, 1.745) |
| 24–27.9 | .16 | 0.719 | (0.453, 1.139) | .078 | 1.503 | (0.955, 2.364) |
| ≥28 | .65 | 0.788 | (0.282, 2.204) | .44 | 1.482 | (0.550, 3.988) |
| Unknown | Ref | _ | _ | Ref | _ | _ |
| **Diseases stage at baseline** |  |  |  |  |  |  |
| Ⅰ | .573 | 0.748 | (0.272, 2.056) | .31 | 1.441 | (0.708, 2.931) |
| Ⅱ | .624 | 0.776 | (0.282, 2.138) | .36 | 1.392 | (0.685, 2.826) |
| Ⅲ | .528 | 0.724 | (0.266, 1.971) | .27 | 1.484 | (0.738, 2.981) |
| Ⅳ | .464 | 0.688 | (0.253, 1.872) | .2 | 1.587 | (0.783, 3.211) |
| **AST at baseline** |  |  |  |  |  |  |
| <20 U/L | .017 | 0.54 | (0.327, 0.894) | .0051 | 1.937 | (1.219, 3.075) |
| 20～30 U/L | .151 | 0.712 | (0.447, 1.132) | .053 | 1.519 | (0.995, 2.319) |
| 30～40 U/L | 1 | 1 | (0.617, 1.62) | .67 | 1.098 | (0.711, 1.694) |
| Missing | Ref | _ | _ | Ref | _ | _ |
| **ALT at baseline** |  |  |  |  |  |  |
| <20 U/L | .714 | 594.954 | (0, 3.9E+17) | 0 | 0.000 | (9.1E-5, 0.0006) |
| 20～30 U/L | .718 | 542.328 | (0, 3.5E+17) | 0 | 0.000 | (9.8E-5.0006) |
| 30～40 U/L | .701 | 791.726 | (0, 5.2E+17) | 0 | 0.000 | (6.7E-5, 0.0004) |
| Missing | Ref | _ | _ | Ref | _ | _ |
| **TBIL at baseline** |  |  |  |  |  |  |
| <10μmol/L | .297 | 1.262 | (0.815, 1.955) | .16 | 0.754 | (0.510, 1.113) |
| 10～20μmol/L | .725 | 1.084 | (0.691, 1.702) | .47 | 0.863 | (0.576, 1.291) |
| Missing | Ref | _ | _ | Ref | _ | _ |
| **CD4 cell count at baseline** |  |  |  |  |  |  |
| <200/μL | .46 | 0.853 | (0.558, 1.302) | .48 | 1.163 | (0.763, 1.769) |
| 200～349/μL | .611 | 1.093 | (0.776, 1.539) | .55 | 0.902 | (0.644, 1.263) |
| ≥350/μL | Ref | _ | _ | Ref | _ | _ |
| **CD8 cell count at baseline** |  |  |  |  |  |  |
| <800/μL | .819 | 1.04 | (0.742, 1.457) | .86 | 0.970 | (0.699, 1.344) |
| 800～1499/μL | .595 | 0.921 | (0.679, 1.248) | .74 | 1.051 | (0.784, 1.407) |
| ≥1500/μL | Ref | _ | _ | Ref | _ | _ |
| **CD4/CD8 Ratio** **at baseline** |  |  |  |  |  |  |
| <0.3 | .028 | 0.524 | (0.295, 0.931) | .033 | 1.818 | (1.049, 3.148) |
| 0.3～0.59 | .07 | 0.604 | (0.35, 1.041) | .079 | 1.602 | (0.946, 2.709) |
| ≥0.60 | Ref | _ | _ | Ref | _ | _ |
| **Cytomegalovirus infection** | .074 | 1.915 | (0.939, 3.908) | .44 | 0.544 | (0.115, 2.560) |

Table S5. CD4/CD8 ratio recovery difference among the three ART treatment subgroups.

| Variable | LPV-based | EFV-based | NVP-based | χ2 | *P* |
| --- | --- | --- | --- | --- | --- |
| Total | 205 | 1415 | 820 |  |  |
| CD4/CD8 ratio recovered | 20 | 140 | 83 |  |  |
| CD4/CD8 ratio unrecovered | 185 | 1275 | 737 |  |  |
| CD4/CD8 ratio recovered rate | 9.8% | 9.9% | 10.1% | .04 | .98 |
| Abbreviations: LPV, Lopinavir; EFV, Efavirdine ; NVP, Nevirapine. | | | | | |

Supplementary Table 6. The liver damage rate of HIV patients receiving ART, group by ART regimen.

|  | Total patients  n | Liver damage  n(%) | Person years | Liver damage  /100 person years | *P* |
| --- | --- | --- | --- | --- | --- |
|  |  |  |  |  |  |
| **LPV-based** |  |  |  |  |  |
| recovered | 20 | 1(0.05) | 127.83 | 0.78 | 0.11 |
| unrecovered | 185 | 30(0.16) | 616.58 | 4.87 |  |
| **EFV-based** |  |  |  |  |  |
| recovered | 140 | 7(0.05) | 822.75 | 0.85 | <0.001 |
| unrecovered | 1275 | 212(0.17) | 4033.75 | 5.26 |  |
| **NVP-based** |  |  |  |  |  |
| recovered | 83 | 4(0.05) | 770.58 | 0.52 | <0.001 |
| unrecovered | 737 | 237(0.32) | 3592.42 | 6.60 |  |

Abbreviations: HIV, human immunodeficiency virus; ART, antiretroviral treatment; LPV, Lopinavir; EFV, Efavirdine ; NVP, Nevirapine.
